# A Super Enhancer-Derived Enhancer RNA Acts Together with CTCF/Cohesin in Trans to Regulate Erythropoiesis

**DOI:** 10.3390/genes16040389

**Published:** 2025-03-28

**Authors:** Bingning Xie, Ann Dean

**Affiliations:** Laboratory of Cellular and Developmental Biology, National Institute of Diabetes and Digestive and Kidney Diseases, National Institutes of Health, Bethesda, MD 20892, USA

**Keywords:** eRNA, erythropoiesis, trans acting, CTCF/cohesin

## Abstract

**Background/Objectives**: Enhancer RNAs (eRNAs) function in diverse modes and increasing studies have shown that they play important roles in normal development and disease. However, their role in erythropoiesis is not fully understood. **Methods**: We analyzed published RNA-seq and Promoter Capture Hi-C data from mouse E14.5 fetal liver cells to identify enhancer RNAs in erythroid cells with long-range interactions. **Results**: We discovered an erythroid-specific enhancer RNA (*CpoxeRNA*) transcribed from an enhancer region upstream of *Cpox*, an enzyme important for heme synthesis. *CpoxeRNA* is important for erythropoiesis, as the knockdown of *CpoxeRNA* by shRNA results in impaired enucleation and cell proliferation during terminal differentiation. *CpoxeRNA* interacts with cohesin and acts both *in cis* and *trans* to regulate erythroid genes. **Conclusions**: we have identified a *trans-acting* eRNA, *CpoxeRNA*, as a potential regulator of terminal erythropoiesis.

## 1. Introduction

Active enhancers can undergo bidirectional transcription [1,2], producing non-coding RNAs known as enhancer RNAs (eRNAs) [1,3,4], which typically have short half-lives [5]. However, several studies have reported that some eRNAs play roles in regulating target gene expression [6,7,8,9,10]. The modes of action of eRNAs include (1) altering the chromatin environment and enhancing chromatin accessibility to allow more access for transcription factors and other regulatory proteins to bind [11]; (2) facilitating enhancer–promoter interactions [9,12]; (3) recruiting regulators to facilitate transcription, including chromatin-modifying enzymes, cohesin, transcription factors, coactivators, etc. [9,13,14,15,16,17,18,19,20]; and (4) facilitating RNAPII pause–release [10,21]. eRNAs may act in cis to regulate neighboring genes or act in trans, diffusing within the nucleus to regulate genes across the genome [22,23]. The regulation of erythropoiesis involves numerous non-coding RNAs [24,25,26,27], for example, lncRNA (UCA1) functions as a scaffold to recruit PTBP1 to stabilize ALAS2 mRNA [28]. A loss of UCA1 will inhibit heme synthesis and block erythroid differentiation at the proerythroblast stage [28]. Trans-acting eRNAs were also reported to participate in regulating erythropoiesis [29,30]. Although many non-coding RNAs have been reported to be involved in erythropoiesis regulation, a comprehensive understanding is still lacking.

Cohesin and CTCF have both been reported to interact with RNA [9,23,31,32,33,34,35,36]. Chromatin looping established by cohesin and CTCF is reduced upon RNA depletion, either through RNase treatment or eRNA knockdown [9,35,36]. eRNAs have also been reported to recruit the CTCF/cohesin complex to target gene loci, thereby activating their transcription [23]. However, the interaction between CTCF and RNA is controversial. While some studies suggest that RNA binding is essential for CTCF’s role in gene expression and chromatin organization [35,36], Guo et al. argued that RNA binding by CTCF is a technical artifact [37], although this conclusion may stem from data misanalysis [38]. Nevertheless, the detailed mechanism through which the CTCF/cohesin complex may interact with eRNAs and the functions of their interaction on gene expression are not fully understood.

We analyzed published RNA-seq data from mouse E14.5 fetal liver cells, integrating this with erythroid enhancer information and Promoter Capture HiC data to identify enhancer RNAs with long-range interactions that are expressed in erythroid cells. Among these erythroid-specific, long-range interacting eRNAs, we identified one, *CpoxeRNA*, derived from a super enhancer also containing the *Cpox* gene. The knockdown of *CpoxeRNA* by shRNA results in impaired proliferation and enucleation during terminal erythropoiesis. Additionally, *CpoxeRNA* plays an architectural role in mediating chromatin looping between its genomic locus and TAD boundaries, as these interactions are reduced upon *CpoxeRNA* loss. We found that *CpoxeRNA* interacts with the CTCF/cohesin complex, migrating to other chromatin loci to regulate genes important for erythropoiesis.

## 2. Materials and Methods

### 2.1. Cell Culture

MEL cells were cultured in Dulbecco’s modified Eagle’s medium (DMEM) supplemented with 10% fetal bovine serum in a humidified incubator with 5% CO_2_. The differentiation of MEL cells was induced by 2% Dimethylsulfoxide for 5 days. C2C12 cells were cultured in DMEM medium supplemented with 20% fetal bovine serum in a humidified incubator with 5% CO_2_.

### 2.2. Mouse E14.5 Fetal Liver Cell Ex Vivo Differentiation

C57BL6 mice were purchased from Jakson Laboratory. All animal studies were performed in accordance with guidelines of the Animal Care and Use Committee of the National Institutes of Health under a Division of Intramural Research, NIDDK-approved animal study protocol (K042LCDB19). Mouse fetal liver cells were purified from E14.5 fetal livers and cultured and differentiated according to the protocol from Dr. Hu’s lab. The medium used for the expansion of erythroid progenitors was StemSpan SFEM II (StemCell Technologies, Vancouver, BC, Canada) containing 100 ng/mL recombinant mouse SCF (Amgen, Thousand Oaks, CA, USA), 40 ng/mL recombinant human IGF-1 (R&D Systems, Minneapolis, MN, USA), 100 nM dexamethasone, and 2 U/mL EPO (Amgen). The medium used to induce differentiation of erythroid progenitors was Iscove-modified Dulbecco’s medium containing 25% FBS (StemCell Technologies), 500 μg/mL holo-transferrin (Sigma-Aldrich, St. Louis, MA, USA), 2 mM L-glutamine, 10 μg/mL recombinant human insulin (Sigma-Aldrich, St. Louis, MA, USA), and 0.5 U/mL EPO.

### 2.3. 5′- and 3′-Rapid Amplification of the cDNA Ends (5′-RACE and 3′-RACE)

5′- and 3′-RACE were performed using the classic RACE method [39,40]. Q5^®^ High-Fidelity 2X Master Mix (NEB, Ipswich, MA, USA, catalog number: M0492) was used for the PCR experiment. See Supplementary Table S1 for primers.

For 5′- RACE, 5 μg of total RNA was used for reverse transcription, with gene-specific reverse-transcription primer and SuperScript™ II Reverse Transcriptase (ThermoFisher, Waltham, MA, USA, catalog number: 18064014). We diluted the reaction mixture to 400 μL with TE (pH 7.5), then purified the cDNA with the miniElute PCR kit (QIAGEN, Hilden, Germany, catalog number: 28004) and eluted it with 16 μL H_2_O. Then we proceeded with the tailing reaction with Terminal Transferase (NEB, Ipswich, MA, USA, catalog number: M0315S), 2 μL of 10× TdT buffer, 2 μL of 2.5 mM CoCl_2_, 4 μL of 1 mM dATP, and 0.5 μL of TdT and added the cDNA and H_2_O to a final volume of 20 μL. The reaction without TdT was used as the mock. We then incubated the tailing reaction at 37 °C for 20 min and 70 °C for 10 min and then diluted it to 500 μL with TE buffer. The 5′-RACE 1st round PCR program was as follows: 98 °C 5 min, 48 °C 2 min, 72 °C 40 min, [98 °C 10 s, 66 °C 10 s, 72 °C 2 min, 30 cycles], 98 °C 10 s, 66 °C 10 s, 72 °C 15 min, and 12 °C ∞. The 5′- RACE 2nd round PCR program was as follows: 98 °C 5 min, [98 °C 10 s, 66 °C 10 s, 72 °C 2 min, 30 cycles], 98 °C 10 s, 66 °C 10 s, 72 °C 15 min, and 12 °C ∞.

For 3′- RACE, 5 μg of the total RNA was reverse transcribed with the Q_T_ primer using SuperScript™ II Reverse Transcriptase (ThermoFisher, Waltham, MA, USA, catalog number: 18064014). The 1st round and 2nd round PCR programs were as follows: 98 °C 30 s, [98 °C 10 s, 66 °C 30 s, 72 °C 5 min, 30 cycles], 98 °C 10 s, 66 °C 30 s, 72 °C 15 min, and 12 °C ∞.

### 2.4. RNA Fluorescent in Situ Hybridization (FISH)

Probes were generated by labeling the 1 nmol oligo mixture with TdT buffer 300 nM dye-ddUTP, 1× TdT, and 0.25 mM CoCl_2_ at 37 °C overnight. After overnight incubation, the probes were precipitated with NaOAc and linear acrylamide (ThermoFisher, Waltham, MA, USA, catalog number AM9520). Cells were fixed with 4% formaldehyde at room temperature for 10 min, washed with PBS twice, had 70% ethanol added, and stored at 4C overnight. The fixed cells were centrifuged to remove the ethanol, then resuspended in 1 mL of wash buffer (2× SSC, 10% Formamide), left to stand for 5 min, and centrifuged to remove the buffer. We then added 100 μL of the hybridization buffer (1 g of Dextran sulfate, 10 mg of *E. coli* tRNA, 100 μL of the 200 mM Vanadyl ribonucleoside complex (NEB, Ipswich, MA, USA, catalog number: S1402S), 40 μL of 50 mg/mL RNase-free BSA, and 1 mL of 20× SSC, 1 mL deionized Formamide (ThermoFisher, Waltham, MA, USA, catalog number: AM9342) to nuclease-free water with a 10 mL final volume) containing probes, incubated it in the dark overnight at 30 °C, washed it with 1 mL of the wash buffer, resuspended it in 1 mL of the wash buffer, and incubated it at 30 °C for 30 min. The pellet was then resuspended in another wash buffer containing 5 ng/mL DAPI and incubated at 30 °C for 30 min. We removed the buffer and resuspended it in the anti-bleach buffer without enzymes (100 μL of 20× SSC, 40 μL of 10% glucose, and 5 μL of 2M Tris-HCl in 0.85 mL of nuclease-free water) for 2 min, and then removed the buffer and resuspended it in 100 μL of anti-bleach buffer with 1 μL of glucose oxidase (Sigma Aldrich, St. Louis, MA, USA, catalog number: G2133-10KU) and 1 μL of catalase (Sigma Aldrich, St. Louis, MA, USA, catalog number: C3515-10MG) before imaging.

### 2.5. shRNA Knock Down

shRNA was designed with tools from the Broad Institute (https://portals.broadinstitute.org/gpp/public/seq/search accessed on 16 January 2025) and cloned into a pLKO.1-puro vector, then electroporated into MEL cells with BTXpress High-Performance Electroporation Kit & Solution (BTXpress, Holliston, MA, USA, catalog no. 89130-538) and the BioRad instrument. See Supplementary Table S1 for the shRNA sequence. shRNA knock down in fetal liver cells were performed with packaging retrovirus with pWH64 and shRNA containing pWH99 plasmids then spinfection to ex vivo cultured fetal liver cells.

### 2.6. Flow Cytometry Assays

Flow cytometry experiments were conducted on the FACS BD LSRFortessa machine (BD Biosciences). Cells were immunostained with PE Rat Anti-Mouse CD71 (BD Biosciences, Catalog #:553267) and allophycocyanin-conjugated anti-TER119 (BD Biosciences, Becton Drive Franklin Lakes, NJ, USA, Catalog #:557909) antibodies, as well as 5 μg/mL Hoechst 33342 (Thermofisher, Waltham, MA, USA, Catalog #: 62249) [41,42].

### 2.7. CRISPR-Cas9 Knock out

sgRNAs were designed using CRISPick (Broad institute, MIT, Cambridge, MA, USA) and cloned into pSpCas9(BB)-2A-Puro (PX459) V2.0 (pSpCas9(BB)-2A-Puro (PX459) V2.0 was a gift from Feng Zhang (Addgene plasmid # 62988; http://n2t.net/addgene:62988 (accessed on 27 May 2021); RRID: Addgene_62988)) according to the protocol of Ran F., et al. [43]. sgRNA plasmids were electroporated into MEL cells with the BTXpress High-Performance Electroporation Kit & Solution (BTXpress, Holliston, MA, USA, catalog no. 89130-538). Cells were transferred immediately into the DMEM medium with 20% FBS and cultured for one day, then treated with 4 μg/mL puromycin for 12 h and washed and cultured in puromycin-free DMEM with 10% FBS. The selection of single colonies via the serial dilution culture was performed in 96-well plates. The genotyping of single colonies was performed after the extraction of DNA with the QuickExtract DNA Extraction Solution (Biosearch Technologies, Teddington TW11 0LY, UK, catalog no. 76081-766) and PCR amplification with the Q5^®^ High-Fidelity 2X Master Mix (NEB, Ipswich, MA, USA, catalog no. M0492S). PCR products were confirmed by Sanger sequencing (Genewiz, Freiburg, Germany).

### 2.8. Subcellular Fractionation

iMEL cells were collected via spinning in a pre-chilled centrifuge at 300× *g* for 5 min and washed with cold PBS, then cells were lysed on ice for 10 min with lysis buffer (20 mM HEPES-KOH, pH 7.5, 80 mM KCl, 15 mM MgCl_2_, 1% Triton X-100, 1× Protease Inhibitor, 2 mM DTT, SuperaseIN, Thermo Fisher Scientific, Waltham, MA, USA). During this incubation, the lysate was gently passed through a pre-chilled, clean 27-gauge syringe 4 times. We also saved 100 µL of the lysate as the “T” (total RNA) fraction. We centrifuged the rest of the cell lysate for 5 min at 700× *g*, 4 °C to pellet nuclei. We then transferred the supernatant (~200 µL) to new tubes as the “C” (Cytoplasmic RNA) fraction. Then, we resuspended the nuclei in 100 µL of DEPC-treated H_2_O. We immediately added 10× volume of TRIzol to the lysate and continued RNA extraction following the TRIzol protocol.

### 2.9. RT-qPCR

RNA was isolated with the RNeasy Plus Mini Kit (QIAGEN, Hilden, Germany, catalog no. 74134). Next, 2 μg of RNA was reverse-transcribed into cDNA by the SuperScript™ III First-Strand Synthesis System (Thermofisher, Waltham, MA, USA, catalog no.18080051), and then analyzed by qPCR with the iTaq Universal SYBR Green Supermix (Biorad, Hercules, CA, USA, catalog no. 1725124) using a QuantStudio 6 FLEX machine (Thermofisher, Waltham, MA, USA). See Supplementary Table S1 for primers. Actin was used as an internal control gene.

### 2.10. ChIP-qPCR

The RAD21 antibody (Abcam, Cambridge, UK, ab992) and MEL cells differentiated for 5 days in 2% DMSO were used for Chromatin immunoprecipitation as previously described [44]. Eluted DNA was analyzed by qPCR with the iTaq Universal SYBR Green Supermix (Biorad, Hercules, CA, USA, catalog no. 1725124) and the QuantStudio 6 FLEX machine (Thermofisher, Waltham, MA, USA). See Supplementary Table S1 for primers.

### 2.11. fCLiP-qPCR

MEL cells differentiated for 5 days in 2% DMSO were used for fCLiP as previously described [45]. Eluted RNA was reverse-transcribed into cDNA using the SuperScript™ III First-Strand Synthesis System (Thermofisher, Waltham, MA, USA, catalog no.18080051) and then analyzed via qPCR with the iTaq Universal SYBR Green Supermix (Biorad, Hercules, CA, USA, catalog no. 1725124) and the QuantStudio 6 FLEX machine (Thermofisher, Waltham, MA, USA). The RAD21 antibody (Abcam, Cambridge, UK, ab992) was used for immunoprecipitation. See Supplementary Table S1 for primers.

### 2.12. RNA Pull-Down Experiment

The interaction between *CpoxeRNA* and the CTCF/cohesin complex was examined using the Pierce™ Magnetic RNA-Protein Pull-Down Kit (ThermoFisher, Waltham, MA, USA, Catalog number20164). Briefly, a primer pair targeting the non-repetitive part of *CpoxeRNA* was used for in vitro transcription. The resulting RNA and the two control RNAs included in the RNA-Protein Pull-Down Kit were labeled according to the manual. A custom-prepared Pierce IP Lysis Buffer (25 mM Tris•HCl pH 7.4, 150 mM NaCl, 1% NP-40, 1 mM EDTA, and 5% glycerol) was used for cell lysis. The steps were performed according to the manual. For the RNaseA-treated sample, RNaseA with a 10 μg/mL final concentration was used.

### 2.13. ChIRP Sequencing

A total of six 20-mer DNA probes targeting *CpoxeRNA* were designed using the single-molecule FISH online designer (https://www.biosearchtech.com/products/rna-fish/chirp-probe-sets (accessed on 20 May 2020)) and synthesized probes from IDT with 3′-biotin-TEG modification. MEL cells differentiated in 2% DMSO containing the DMEM medium for 5 days were used for the ChIRP experiments. Fifty million cells per sample were crosslinked with 3% formaldehyde at room temperature for 30 min. Cells were sonicated in the ChIRP lysis buffer with the probe sonicator (Branson SFX150 Sonifier, Emerson, St. Louis, MO, USA) with 30 s ON, 45 s OFF, and 30 min total ON time at 38% J. The steps were performed according to the protocol [46]. Commercial lacZ probes (Magna ChIRP™ Negative Control Probe Set, Millipore Sigma, St. Louis, MA, USA, Catalog #: 03-307) were used as control probes. RNA and DNA pull-down efficiency was confirmed by qPCR. The sequencing library was prepared with the NEBNext^®^ Ultra™ II DNA Library Prep Kit for Illumina and sequenced with the NovaSeq 6000 system by the National Heart, Lung, and Blood Institute (NHLBI) DNA Sequencing and Genomics Core.

### 2.14. 3C-qPCR

MEL cells differentiated for 5 days in 2% DMSO were used for 3C experiments, performed as previously described [47,48]. DNA was analyzed by qPCR with the iTaq Universal SYBR Green Supermix (Biorad, Hercules, CA, USA, catalog no. 1725124) and the QuantStudio 6 FLEX machine (Thermofisher, Waltham, MA, USA,). BAC controls were obtained from BACPAC company; alpha *Aortic Actin*-2(RP23-2N15), *Dcbld2*-*Cpox* locus (RP23-317L24, RP24-264K24, RP24-371018). See Supplementary Data for primers. Samples were diluted 10x when detecting the *Dcbld2* promoter and diluted 3× when detecting the *Cpox* promoter. Consequently, the deltaCt values for the *Dcbld2* promoter and the *Cpox* promoter were subtracted with log2(10) and log2(3) before the downstream calculation steps.

### 2.15. Data Analysis

#### 2.15.1. Analysis of Public Datasets

Raw RNA-seq data from Erythroid cells (ENCODE mouse E14.5 fetal liver cells ENCSR000CHE [49], Ter119-positive cells [24,50], and Ter119-negative cells [24]) were mapped to the mm9 genome using STAR. The transcriptome was assembled by StringTie. Then, we used Gffcompare to select transcripts with class codes of “x”, “i”, “u”, and “r”. The Bedtools intersect function was used to find the transcript that overlapped with the erythroid enhancer. Coding transcripts were removed by CPC2 [51]. Promoter-Capture HiC data from mouse E14.5 fetal liver cells [52] were analyzed with GenomicInteractions [53] to select eRNAs with long-range interactions (>50 kb). The resulting 791 eRNAs were further filtered for erythroid-specific eRNAs by analyzing ENCODE mouse RNA-seq data with salmon [54] (supplied with a custom reference file, which combined GENCODE M21 and eRNA sequences) and TissueEnrich [55] (see Supplementary Table S2 for the full list of datasets used for analysis). RNA-seq data from BFU, CFU, and Ter119+ cells were analyzed with salmon [54], then DEseq2 [56] was used to find differentially expressed genes.

RNA-seq data for BFU, CFU, and Ter119+ cells were obtained from GSE52126 [24] and GSE26086 [50]. Promoter-Capture HiC data (“FLC_promoter_other_significant_interactions.txt”) from Schoenfelder et al. [52] were downloaded from ArrayExpress (accession E-MTAB-2414) in 2019. Erythroid-specific enhancers are a union set of enhancers from ENCODE3 mouse E14.5 fetal liver cells [57] and enhancers in MEL, G1E, and G1ER cells annotated by Enhancer atlas 2.0 [58].

Mouse E14.5 fetal liver, ESC, and C2C12 cell HiC data were analyzed by Juicer [59] and visualized with Juicebox [60]. The raw Fastq files for E14.5 fetal liver and ESC were downloaded from ArrayExpress (accession E-MTAB-2414 [52]), while the raw Fastq files for C2C12 (Myoblasts) were downloaded from Doynova M., et al. [61] GSE84279.

ENCODE ChIP-seq data: RAD21 (ENCSR000ETS), CTCF (ENCSR000ETQ), and SMC3 (ENCSR000ETL).

#### 2.15.2. Gene Ontology Enrichment Analysis

Gene Ontology analysis of the genes interacting with erythroid enhancers was performed using the DAVID database [62] and plotted with ggplot2.

#### 2.15.3. ChIRP Sequencing Data Analysis

Raw reads were trimmed with Trim Galore (v0.6.7) and aligned to the mm10 genome using Bowtie2 (v 2.5.3) [63]. Peaks were called for each probe set and replicated using the callpeak function from MACS2 [64] relative to the input. The resulting peaks within 1 kb regions were merged using the Bedtools merge function, the merged peaks were extended by 2 kb with the slope function in Bedtools [65], and common peaks among all the replicates from EVEN and ODD probe sets were found using pybedtools [66]. The peak distributions were analyzed using the ChIPseeker package [67]. A Circos plot was plotted with shinyCircos (v2.0) [68]. ChIRP-seq data were deposited into the Gene Expression Omnibus (accession no. GSE288876).

## 3. Results

### 3.1. Identification of Enhancer RNAs Expressed in Erythroblasts Involved in Long-Range Interactions

**Figure 1:**
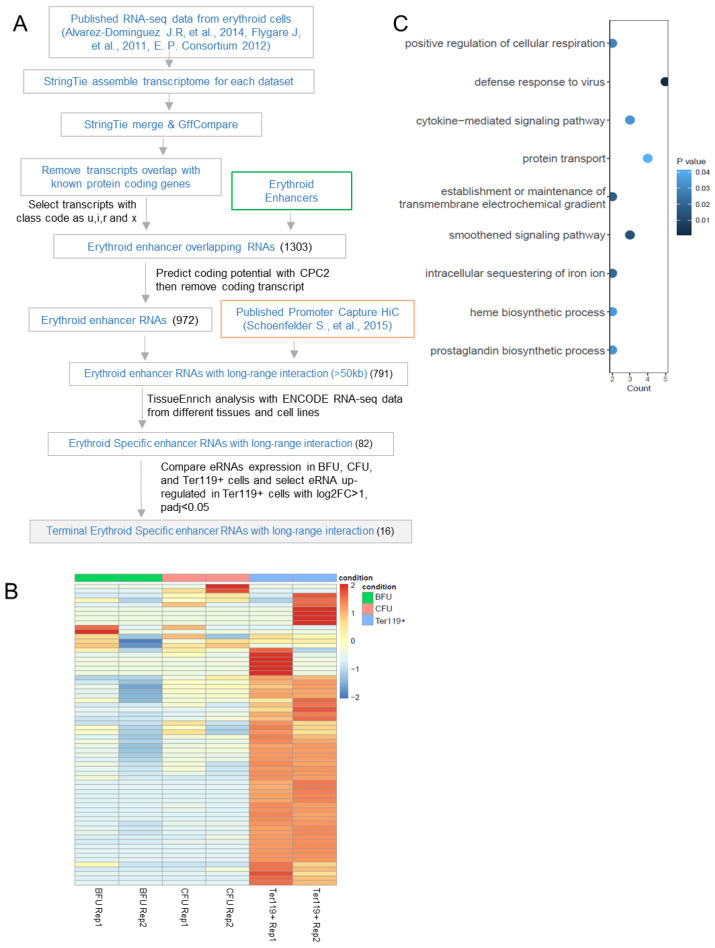
Identification of enhancer RNA expressed in the erythroblast with long-range interactions. (**A**) Workflow showing the discovery of enhancer RNAs in erythroid cells with long-range interactions, published data from [24,49,50,52] were used for analysis. (**B**) Heatmap showing the expression of the long-range interacting erythroid enhancer RNA in BFU, CFU, and Ter119-positive cells from mouse E14.5 fetal liver. RNA-seq data from Flygare J., et al. [50]. (**C**) Gene ontology analysis of the target genes that interact with Ter119 cells’ specific long-range interacting erythroid enhancer RNAs.

To identify erythroid-specific enhancer RNAs (eRNAs) with long-range interactions, we analyzed previously published data by using the strategy outlined in Figure 1A. We used RNA-seq data from a mouse E14.5 fetal liver [24,49,50], which is essentially an erythroid organ, and assembled the transcriptome using StringTie [69]. To identify novel eRNA transcripts, we applied GffCompare to the assembled transcriptome, removed transcripts overlapping known protein-coding genes, and further selected transcripts with class codes “x”, “u”, “I”, and “r”. We then selected transcripts overlapping mouse erythroid enhancers using Bedtools [65]. The mouse erythroid enhancer dataset employed is a union of enhancers from ENCODE3 mouse E14.5 fetal liver cells [57] and enhancers annotated in MEL, G1E, and G1ER erythroid cell lines by EnhancerAtlas 2.0 [58]. Transcripts with coding potential were removed using the Coding Potential Calculator (CPC2) [51], and as a result, 972 eRNAs were identified.

We then used published Promoter Capture HiC data from mouse E14.5 fetal liver cells [52] to select from these 972 eRNAs those with long-range interactions (>50 kb), obtaining 791 such eRNAs. To further identify erythroid-specific long-range interacting eRNAs, we analyzed the tissue/cell expression patterns of these 791 long-range interacting eRNAs using 28 total RNA-seq datasets and 56 polyA RNA-seq datasets from ENCODE with classification performed using TissueEnrich [55]. The resulting 82 erythroid-specific eRNAs commonly identified among all datasets were defined as erythroid-specific long-range interacting eRNAs.

We next examined the expression patterns of these erythroid-specific long-range interacting eRNAs across erythroid lineages, including burst-forming units (BFU), colony-forming units (CFU), and Ter119+-maturing erythroblasts [50]. The majority of eRNAs were induced in erythroblasts, suggesting a role in terminal erythropoiesis (Figure 1B). We performed differential expression analysis with DEseq2 and identified 16 eRNAs that were significantly upregulated in Ter119+ cells. Gene ontology analysis of the 53 genes interacting with these 16 eRNAs loci shows enrichment for the heme biosynthetic process (Figure 1C). These results show that while a few of the erythroid eRNA loci with long-range interactions that we identified as being upregulated in late erythropoiesis make contact with erythroid genes, many do not. This implies that if these eRNAs function in late erythropoiesis, they may act indirectly or regulate erythroid genes in trans without relying on chromatin looping.

### 3.2. CpoxeRNA Is an Erythroid-Specific Enhancer RNA

**Figure 2:**
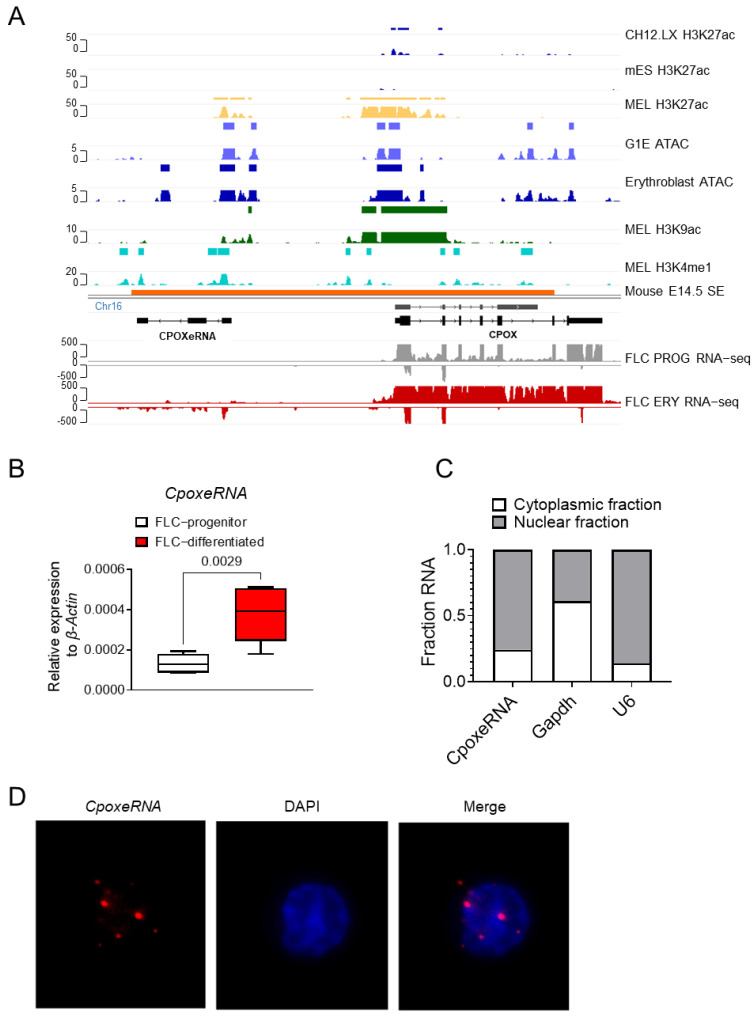
*CpoxeRNA* is an erythroid-specific enhancer RNA. (**A**) IGV tracks showing the H3K27ac signals in mouse CH12.LX, mouse embryonic stem cell (mES), and MEL cells. H3K9ac and H3K4me1 signals in MEL cells. G1E cell and Erythroblast ATAC signal, Mouse E14.5 super enhancer, and RNA-seq from mouse E14.5 fetal liver erythroid progenitor cells (FLC PROG) and erythroid blast cells (FLC ERY). ChIP-seq data from ENCODE. RNA-seq data from Alvarez-Dominguez et al. [24]. (**B**) qRT-PCR result showing the expression level of *CpoxeRNA* in ex vivo differentiated mouse E14.5 fetal liver cells. Two biological replicates with three technical replicates each, *n* = 6, two-tailed unpaired *t*-test. (**C**) Cell fractionation qRT-PCR result showing the distribution of *CpoxeRNA*. (**D**) Immunofluorescence RNA FISH data showing the localization of *CpoxeRNA*.

Among the eRNAs induced in erythroblasts (Figure 1B), we identified an eRNA transcribed upstream of the *Cpox* gene (Figure 2A). This eRNA is located within the same super enhancer as the *Cpox* gene. ENCODE H3K27ac ChIP-seq data from mouse B cell (CH12.LX), mouse embryonic stem cells (mES) and erythroid cells (MEL), and ATAC-seq data from mouse erythroid cells (G1E) and mouse erythroblasts indicate that this genomic region has chromatin characteristics consistent with being an erythroid-specific enhancer (Figure 2A).

RNA-seq data from mouse E14.5 fetal liver erythroid progenitor cells (FLC PROG) and erythroid cells (FLC ERY) [24] reveal that this eRNA is predominantly expressed from the antisense strand in fetal liver erythroblasts, while a much lower level of transcription occurs from the sense strand (Figure 2A, bottom), consistent with the bidirectional transcription feature of active enhancers [19,70]. In earlier work, we had named this uncharacterized transcript *CpoxeRNA* [71]. The 5′-RACE experiment confirmed the bidirectional transcription of *CpoxeRNA* (Supplementary Figure S1A). A 3′-RACE experiment was conducted to determine the full length of *CpoxeRNA* (Supplementary Figure S1B). Two isoforms of *CpoxeRNA* (1262 nt and 1656 nt) were identified in induced MEL cells with alternative 5′ ends, each containing four exons (Supplementary Figure S1C).

RT-qPCR analysis in both mouse MEL cells [71] and ex vivo differentiated E14.5 mouse fetal liver cells showed that *CpoxeRNA* is induced during terminal erythropoiesis (Figure 2B). Tissue expression analysis further indicates that *CpoxeRNA* is an erythroid-specific eRNA [71]. CPC2 analysis confirms that *CpoxeRNA* is a non-coding RNA (Supplementary Figure S2A), and vertebrate 60-way conservation analysis shows that it is rodent-specific (Supplementary Figure S2B). Cell fractionation RT-qPCR experiments and fluorescence in situ hybridization demonstrate that *CpoxeRNA* is primarily localized in the cell nucleus (Figure 2C,D). Thus, *CpoxeRNA* is a predominantly nuclear, non-coding RNA specific to erythroid cells that is transcribed antisense to the neighboring Cpox gene.

### 3.3. CpoxeRNA Mediates Chromatin Interactions Between Its Genomic Locus and TAD Boundaries

**Figure 3:**
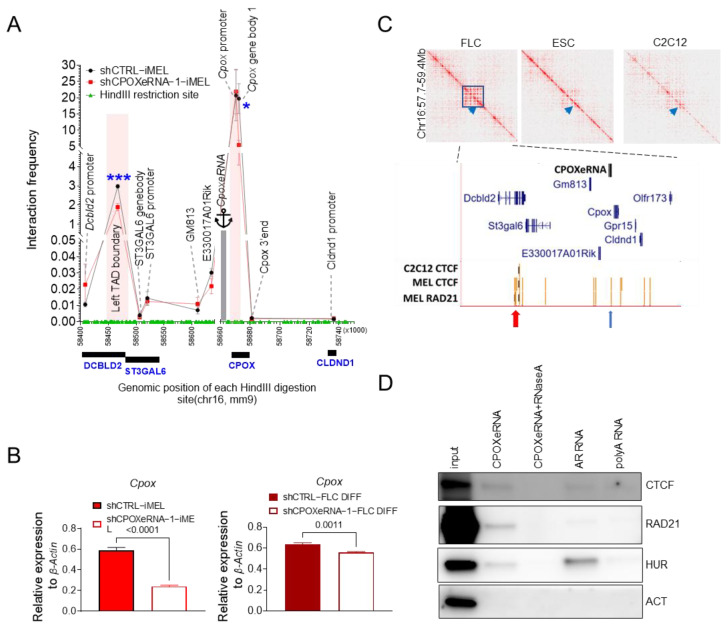
*CpoxeRNA* interacts with CTCF/cohesin complex. (**A**) 3C experiment shows the interactions between *CpoxeRNA* and flanking regions in the control and *CpoxeRNA* knockdown iMEL cells. *CpoxeRNA* locus acts as an anchor. TAD boundary regions are highlighted in red. Three biological replicates with two technical replicates each, *n* = 6, one-tailed unpaired *t*-test. *: *p* ≤ 0.05; ***: *p* ≤ 0.001. (**B**). RT-qPCR results showing *Cpox* expression in the control and shCPOXeRNA knockdown iMEL and ex vivo differentiated E14.5 fetal liver cells. *n* = 3 three technical replicates. Unpaired one-tailed *t*-test. (**C**) HiC maps derived from Mouse E14.5 fetal liver [52], ESC [52], and C2C12 cells [61] showing that *CpoxeRNA* is located in an erythroid-specific TAD. Bottom tracks show the Gene annotation track and ENCODE CTCF narrow peak in C2C12 and MEL cells and RAD21 narrow peak in MEL cells. Red arrows indicate the left TAD boundary and blue arrows indicate the CTCF/RAD21 peaks overlapping with *CpoxeRNA*. (**D**). RNA pull-down experiments showing *CpoxeRNA* interacts with the CTCF/cohesin complex. The *CpoxeRNA* probe with RNaseA treatment (*CpoxeRNA* + RNaseA) was used as the negative control sample. ACT was used as the negative control. AR RNA and polyA RNA were the positive RNA control and negative RNA control for the pull-down experiment procedure, and HUR was used to confirm whether the procedure was working as it binds to AR RNA but not polyA RNA.

PCHiC data [52] show that the *CpoxeRNA* genomic locus interacts with the proximal coding genes *Cpox*, *Gm813,* and *E330017A01Rik* (Supplementary Figure S3A). It also interacts with distal genes *Cldnd1* and *St3gal6*. We performed a 3C experiment and found that *CpoxeRNA* mainly interacts with the two flanking TAD boundaries (Figure 3A). The *CpoxeRNA* genomic locus had the highest interaction frequency with *Cpox*, while for other genes, the interaction frequencies were more than 600-fold lower (Figure 3A).

We next asked whether the *CpoxeRNA* eRNA transcript itself participated in regulating chromatin looping. We performed *CpoxeRNA* knockdown using shRNA in differentiated MEL cells (iMEL) and ex vivo differentiated E14.5 fetal liver cells (Supplementary Figure S3B,C). After the knockdown of *CpoxeRNA*, the interaction frequency of *CpoxeRNA* with the genes located within this TAD (*St3gal6*, *Gm813*, and *E330017A01Rik*) and outside the TAD (*Cldnd1*) were not affected. Surprisingly, we observed that a loss of *CpoxeRNA* significantly decreased the interaction between its genomic locus and both flanking TAD boundaries (Figure 3A).

Since *Cpox* overlaps with the right TAD boundary, we next checked whether its expression was affected after *CpoxeRNA* knockdown, which reduced their interaction. As shown in Figure 3B, the knockdown of *CpoxeRNA* by shRNA in iMEL cells and ex vivo differentiated E14.5 fetal liver cells consistently downregulated *Cpox*. This is consistent with the observation that the *CpoxeRNA* genomic locus is an enhancer of *Cpox* since its deletion using CRISPR-Cas9 (Supplementary Figure S3D) resulted in the downregulation of *Cpox* in both undifferentiated (UMEL) and differentiated MEL (iMEL) cells (Supplementary Figure S3E,F). Together, these results show that *CpoxeRNA*, the eRNA itself, regulates the expression of *Cpox*.

There is a CTCF/cohesin binding site (CBS) located upstream of *CpoxeRNA* (Supplementary Figure S3A). We next asked whether this CBS site plays any role in regulating the activation of *Cpox* by *CpoxeRNA*. We performed deletion of this CBS site using CRISPR-Cas9 (Supplementary Figure S4A,B). In undifferentiated MEL cells (UMEL), the loss of the CBS did not affect *Cpox* expression in one clone and upregulated *Cpox* expression in another clone (Supplementary Figure S4C). However, *Cpox* was upregulated in differentiated MEL cells (iMELs) with the CBS deletion in both clones compared to control cells (Supplementary Figure S4D). These results suggest that the induced expression of *Cpox* is at least partially antagonized by CTCF binding between its promoter and the *CpoxeRNA* genomic location, where it may function as an insulator.

Given that *CpoxeRNA* interacts with TAD boundaries, that the left-side TAD boundary is anchored by the CTCF/cohesin complex, and that there is also a CTCF/cohesin binding site at the *CpoxeRNA* locus (Figure 3C), we next examined whether *CpoxeRNA* can associate with the CTCF/cohesin complex. RNA pull-down and fCLiP-RT-qPCR experiments confirmed that *CpoxeRNA* interacts with the CTCF/cohesin complex (Figure 3D and Supplementary Figure S5A). Following *CpoxeRNA* knockdown, the protein levels of CTCF and RAD21 remained unaffected (Supplementary Figure S5B). However, RAD21 binding at the *CpoxeRNA* genomic locus increased rather than decreased (Supplementary Figure S5C). This suggests that *CpoxeRNA* and its genomic locus compete for RAD21 binding. Upon *CpoxeRNA* depletion, RAD21 can accumulate at its genomic locus. These data support that *CpoxeRNA*, per se, is important for chromatin organization at the *CpoxeRNA*/*Cpox* locus. This also indicates that some of the chromatin loops mediated by CTCF/cohesin depend on RNA.

### 3.4. CpoxeRNA Is Critical for Survival and Proliferation During Terminal Erythropoiesis

**Figure 4:**
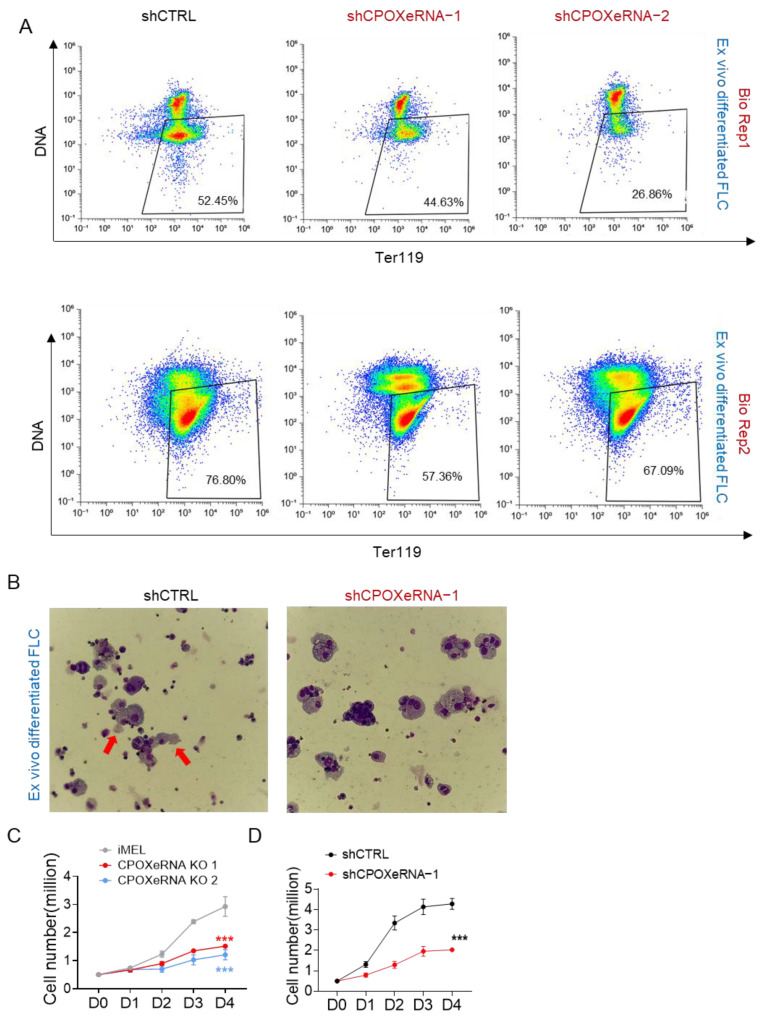
*CpoxeRNA* is critical for survival and proliferation during terminal erythropoiesis. (**A**) The enucleation rate in the control and *CpoxeRNA* knockdown cells. Levels of the differentiation marker TER119 (fluorescent immunolabeling) vs. DNA content (Hoechst staining) of shRNA-transduced cells after 48 h of ex vivo culture were detected by Flow cytometry. (**B**) May–Grunwald Giemsa staining of shRNA-transduced mouse E14.5 fetal liver erythroblasts fixed after 48 h of ex vivo differentiation. Arrow shows enucleated cells. (**C**,**D**) Growth curve shows the cell proliferation rate in the differentiated WT MEL cell (iMEL) after *CpoxeRNA* knockout by CRISPR-Cas9 (**C**) and after transfection with a shRNA target to *CpoxeRNA* (shCPOXeRNA-1) or an empty vector (shCTRL) (**D**). ***: *p* ≤ 0.001.

To investigate whether *CpoxeRNA* plays a role in erythropoiesis, we used shRNA to knock down *CpoxeRNA* in both ex vivo differentiated mouse E14.5 fetal liver cells and differentiated MEL cells (Supplementary Figure S3B,D). We examined erythropoiesis progression in ex vivo differentiated E14.5 fetal liver cells because they can undergo the full erythroid differentiation process, including enucleation. This system provides a more physiologically relevant model for studying erythropoiesis. The depletion of *CpoxeRNA* led to reduced enucleation in ex vivo differentiated fetal liver cells (Figure 4A,B). Additionally, the knockdown of *CpoxeRNA* or knockout of its genomic locus inhibited cell proliferation during MEL cell differentiation (Figure 4C,D). These results indicate that *CpoxeRNA* is essential for the maturation of cells during terminal erythropoiesis.

Together, these results show that, in contrast to the loss of *Cpox* mRNA or its genomic locus inside this super enhancer, which does not affect erythropoiesis [71,72], the loss of eRNA or its genomic locus inside this super enhancer impairs terminal erythropoiesis.

### 3.5. CpoxeRNA Interacts with the CTCF/Cohesin Complex to Act in Trans to Regulate Erythropoiesis

Although *Cpox* encodes the sixth enzyme in the heme synthesis pathway, it appears to be dispensable for erythropoiesis [71,72]. Additionally, other genes that loop with the *CpoxeRNA* locus (Figure 3A and Supplementary Figure S3A) are not directly related to erythropoiesis [71].

**Figure 5:**
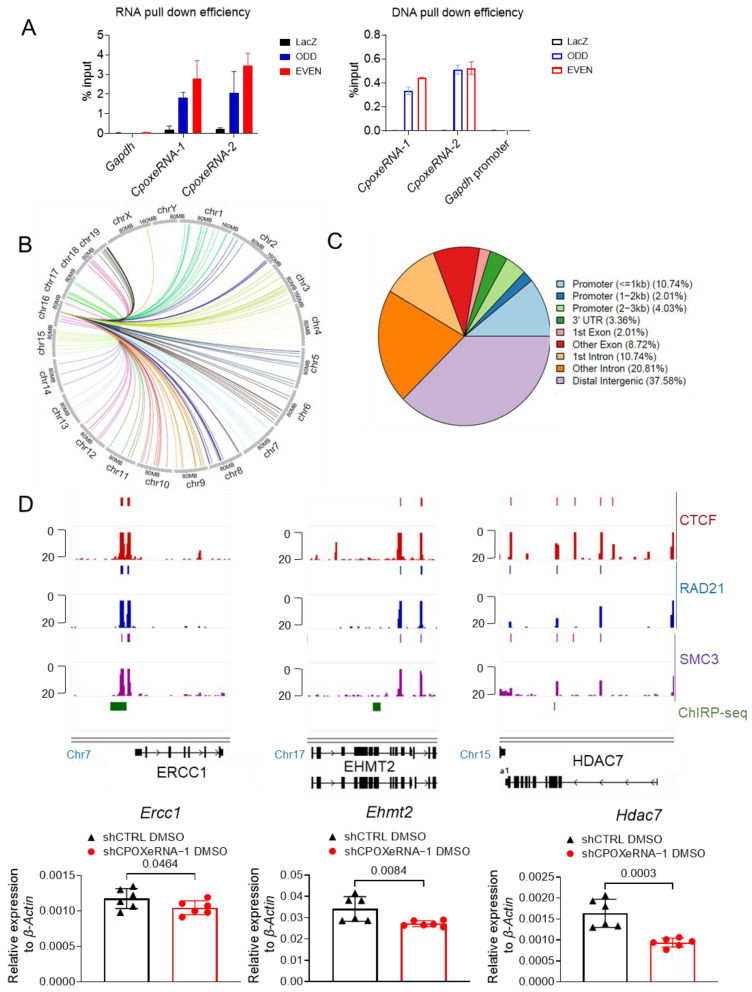
*CpoxeRNA* acts in trans to regulate genes important for erythropoiesis. (**A**) qRT-PCR and qPCR results showing RNA and DNA pull-down efficiency of ChIRP experiment. (**B**) Circos plot showing the genome-wide binding sites of *CpoxeRNA* identified by ChIRP-seq experiment. (**C**) Pie chart showing the genomic distribution of *CpoxeRNA* binding sites identified from ChIRP-seq analysis. (**D**) Top image presents IGV tracks showing the ENCODE ChIP-seq data for CTCF, RAD21, and SMC3 binding at target genes loci in undifferentiated MEL cells and *CpoxeRNA* ChIRP-seq peaks at target genes, while the bottom image presents the qRT-PCR result showing the expression level of target interacting genes identified by the ChIRP experiment in the control and *CpoxeRNA* knockdown cells. Two biological replicates with three technical replicates each (*n* = 6), one-tailed unpaired *t*-test.

Since cohesin can mediate the action of eRNAs in trans to regulate genes on other chromosomes, we investigated whether *CpoxeRNA* might also act in trans. We performed ChIRP-seq with two sets of probes targeting *CpoxeRNA* (Figure 5A). The circos plot illustrates the 149 loci across different chromosomes that are occupied by *CpoxeRNA* (Figure 5B). *CpoxeRNA* occupancy was detected at four sites on chromosome 16 in addition to the *CpoxeRNA* region, but none were within the *CpoxeRNA*/*Cpox* TAD. Most *CpoxeRNA* binding sites are in intergenic regions, although approximately 16% of the 149 loci are in promoter regions (<3 kb) (Figure 5C). We tested 8 genes, and 3 of the 23 genes bound by *CpoxeRNA* at the promoter were downregulated following *CpoxeRNA* knockdown (Figure 5D). These genes are crucial for chromatin remodeling, with *Ercc1* being additionally important for stress erythropoiesis and cell proliferation [73].

In summary, these findings indicate that *CpoxeRNA* interacts with the CTCF/cohesin complex to act in trans, contributing to the regulation of erythropoiesis.

## 4. Discussion

We identified enhancer RNAs (eRNAs) that are enriched in erythroid cells and have long-range interactions by integrating multi-omics approaches. However, we found that the majority of these eRNAs do not form chromatin loops with erythroid genes. This suggests that if these eRNAs play a role in erythropoiesis, they likely act indirectly or function in trans to regulate erythroid genes without being mediated by chromatin looping. Among these erythroid long-range interacting eRNAs, we studied one specific eRNA, *CpoxeRNA*. The knockdown of *CpoxeRNA* by shRNA results in impaired cell proliferation and enucleation during terminal erythropoiesis. Interestingly, in earlier work, we observed that the knockdown of mRNA for the neighboring coding gene *Cpox* had no effect on erythropoiesis [71,72]. Additionally, *CpoxeRNA* plays an architectural role in mediating chromatin looping between its genomic locus and TAD boundaries, as these interactions are reduced upon *CpoxeRNA* loss (Supplementary Figure S5D).

We observed different phenotypes for shRNA KD or KO of *CpoxeRNA*, which inhibit erythropoiesis, compared to KD or KO of *Cpox*, which do not affect erythropoiesis [71]. The latter result is consistent with *Cpox* being dispensable for erythropoiesis [72]. However, *CpoxeRNA* seems important for erythroid cell maturation. We observed impaired proliferation and enucleation after the loss of *CpoxeRNA*. This may be due to other erythroid genes that were dysregulated in the *CpoxeRNA* KD or KO cells, thus affecting terminal erythropoiesis. Indeed, we found that some erythroid gene loci were bound by *CpoxeRNA* via ChIRP-seq experiments, and their expression was reduced upon *CpoxeRNA* loss (Figure 5).

Active enhancers can produce non-coding RNAs, which are often transcribed bidirectionally and have short half-lives due to rapid degradation by the RNA exosome after transcription [3,4,5,34,70]. Studies have reported that some eRNAs play regulatory roles and can activate target promoters that their genomic loci make contact with, acting as RNA activators (RNAa) [9,26,27]. Other studies have reported trans-acting eRNAs that regulate genes located distally on the same chromosome or on different chromosomes, such as Bloodlinc [29], DRReRNA [23], GATA2AS [30], and HOXDeRNA [22]. These types of trans-acting eRNAs are often polyadenylated, allowing them to be protected from degradation upon transcription and potentially enabling them to diffuse within the nucleus [34,70]. We found that *CpoxeRNA* is also polyadenylated, consistent with its proposed trans-acting properties.

Cohesin has been shown to interact with eRNAs, such as DRReRNA, which recruits cohesin to the target gene locus [23,31]. Using the non-repetitive 5′ region of *CpoxeRNA* as bait, we found that it can interact with the CTCF/cohesin complex. The remaining 3′ part of *CpoxeRNA* contains several repetitive elements, including three ERVL family transposons, one of which is rodent-specific and appears to be intact (Supplementary Figure S1C). The role of the 3′ parts of *CpoxeRNA* in its function as an eRNA, and particularly in its mobility, remains unknown.

Our examination of ChIP-seq data from ENCODE shows that the *CpoxeRNA* target gene loci already have CTCF/cohesin binding in undifferentiated MEL cells (Supplementary Figure S3A), while the highest levels of *CpoxeRNA* are found in differentiated MEL cells. There is a CTCF/cohesin binding site in the *CpoxeRNA* locus, which may serve as the source of CTCF/cohesin that binds to *CpoxeRNA* upon its transcription. We did not find inter-chromosomal loops at the *CpoxeRNA* genomic locus in the fetal liver Promoter Capture Hi-C data [52] or high-resolution cell cycle in situ Hi-C data from G1ER cells [74]. Therefore, we propose that *CpoxeRNA* may migrate in the nuclear environment via CTCF/cohesin complex interaction, allowing binding at target sites. However, we cannot rule out the possibility that *CpoxeRNA* is recruited by inter-chromosomal interactions mediated by the CTCF/cohesin complex.

The interaction between cohesin and *CpoxeRNA* is mechanistically different from previously reported cohesin-bound eRNAs, such as ER-regulated eRNAs [9] and DRReRNA [23]. In the case of ER-regulated eRNAs, the loss of these eRNAs leads to decreased cohesin binding at their genomic loci [9], while the knockdown of *CpoxeRNA* resulted in increased RAD21 occupancy at its genomic locus (Supplementary Figure S5C). This suggests that *CpoxeRNA* competes with its genomic locus for cohesin binding and also suggests that some of the chromatin loops mediated by CTCF/cohesin depend on RNA. The underlying mechanism by which CTCF/cohesin and *CpoxeRNA* cooperate to regulate gene expression remains to be further explored.

## Data Availability

The sequencing data generated in this study have been deposited into the GEO database with the accession number GSE288876. Code availability: No original code was generated in this study.

## Supplementary Materials

The following supporting information can be downloaded at: https://www.mdpi.com/article/10.3390/genes16040389/s1, Figure S1: 5′- and 3′-RACE results of *CpoxeRNA*; Figure S2: Coding potential and conservation analysis of *CpoxeRNA*; Figure S3: Knockdown and knockout of *CpoxeRNA*; Figure S4: Deletion of *CpoxeRNA* CBS A; Figure S5: Cohesin interactions with *CpoxeRNA*; Table S1: primer, antibody, and sgRNA info; Table S2: ENCODE total and polyA RNA-seq data info.
