# Supplementary material for: A Super Enhancer-Derived Enhancer RNA Acts Together with CTCF/Cohesin in Trans to Regulate Erythropoiesis
Source: Genes (Basel). 2025 Mar 28;16(4):389. doi: 10.3390/genes16040389 (PMC12026470; doi:10.3390/genes16040389)
Supplement: Supplementary file 1 [file genes-16-00389-s001.zip › genes-3522861-supplementary.pdf]

Supplementary Fig.1 | 5'- and 3'- RACE result of *CpoxeRNA*. A. 5'- RACE results confirm the transcripts bidirectionally transcribed in the *CpoxeRNA* genomic locus. Arrows indicate the correct band. **B.** 3'- RACE confirms the transcript transcribed in the minus strand of *CpoxeRNA* locus. **C.** IGV plot shows the isoforms of *CpoxeRNA*.

Supplementary Fig.2 | Coding potential and conservation analysis of *CpoxeRNA*. A. Predicting coding ability of *CpoxeRNA* with Coding Potential Calculator 2 shows that both isoforms of *CpoxeRNA* are non-coding RNAs. **B.** UCSC genome browser "Vertebrate Multiz Alignment & Conservation" track shows the conservation of *CpoxeRNA* and the RepeatMasker track shows the repetitive elements overlapped with *CpoxeRNA* genomic locus.

Supplementary Fig.3 | **Knock down and knock out of *CpoxeRNA*. A**. UCSC genome browser tracks showing the chromatin loops formed by *CpoxeRNA* genomic locus and the SMC3, RAD21, CTCF binding in the region. Loops detected by PCHiC were shown in blue arches. **B to C.** RT-qPCR results shows the knockdown efficiency of *CpoxeRNA* after shRNA knockdown in differentiated MEL cell (iMEL) (**B**), *ex vivo* differentiated mouse E14.5 fetal liver cell (**C**). Results from three technical replicates for each sample were shown here for knockdown in fetal liver cells (n=3) and shCTRL and shCPOXeRNA-2, and three biological replicates with three technical replicates each for shCTRL and shCPOXeRNA-1 (n=9), unpaired one-tailed Mann-whitney test. **D**. *CpoxeRNA* Knock out confirmation, genotyping PCR result shown on the left and sanger sequencing result shown on the right. **E and F**. RT-qPCR result shows the expression level of *Cpox* in the WT and *CpoxeRNA* KO UMEL (**E**) and iMEL cells (**F**). N=6 (Two biological replicates with three technical replicates each) for UMEL samples, n=3 bilogical replicates for iMEL samples, unpaired one-tailed t test.

Supplementary Fig. 4 | **Deletion of *CpoxeRNA* CBS A.** *CpoxeRNA* promoter CBS Knock out confirmation, genotyping PCR result shown on the left and sanger sequencing result shown on the right. **B.** Diagram of the CTCF deletion at the 3' of *CpoxeRNA*. **C and D.** RT-qPCR result shows the expression level of *CpoxeRNA* and *Cpox* in the WT and CBS deletion UMEL (**C**) and iMEL cells(**D**). N=4 (2 biological replicates with 2 technical replicates each) for UMEL samples, n=3 technical replicates for iMEL samples. One-tailed unpaired t-test. EV: empty vector.

Supplementary Fig. 5 | Cohesin interact with *CpoxeRNA*. A. fCLiP-qRT-PCR result shows the binding of RAD21 with *CpoxeRNA*. N=3 technical replicate, unpaired one-tailed t test. B. Western blot shows the expression level of CTCF and RAD21 in the control and *CpoxeRNA* knockdown cells. **C.** ChIP-qPCR shows the binding of RAD21 at left TAD boundary and *CpoxeRNA* CBS

locus in the control and *CpoxeRNA* knockdown cells. N=3 technical replicates for left TAD boundary and *CpoxeRNA* CBS. One-tailed unpaired t-test. **D**. Mechanistic model of *in cis* and *in trans* action of *CpoxeRNA* in chromatin looping and erythropoiesis regulation.

Supplementary Fig.1

A

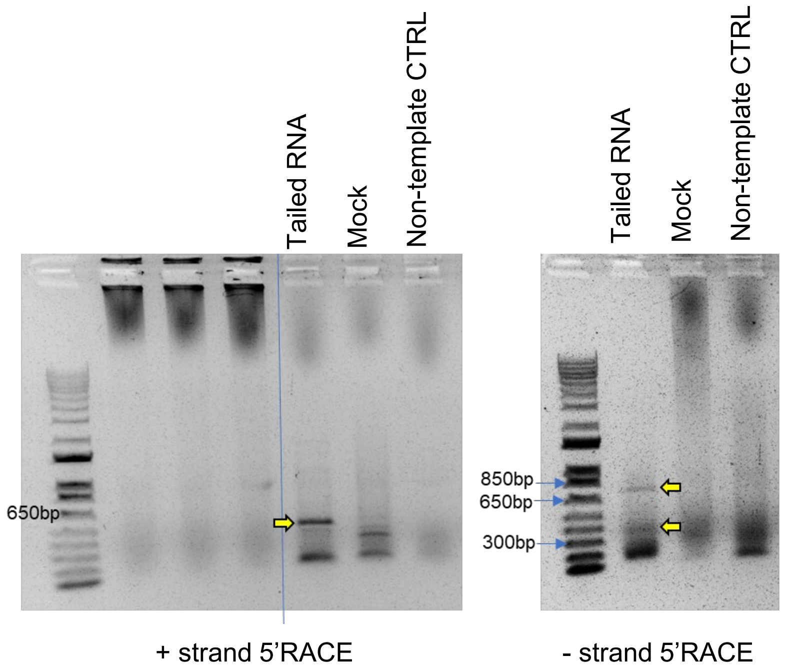

B

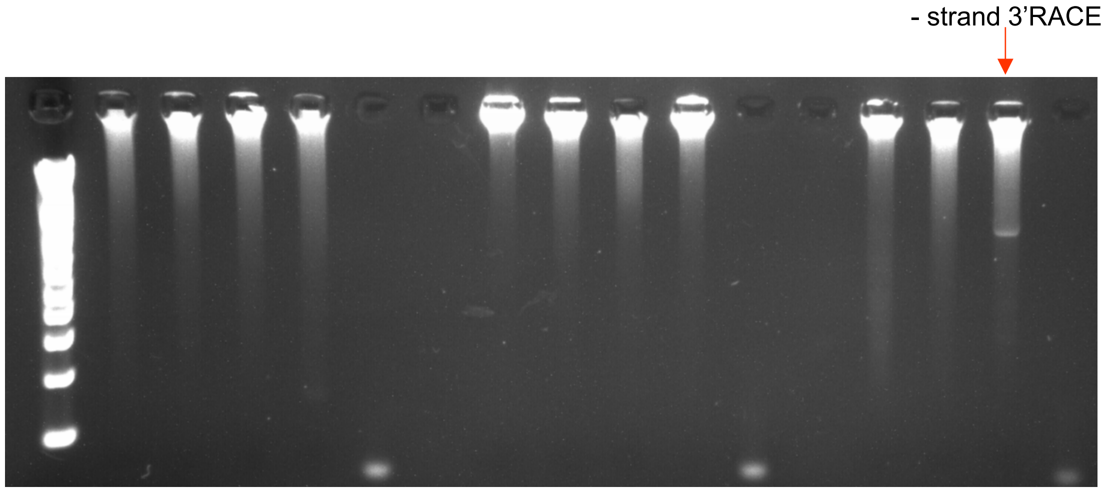

C

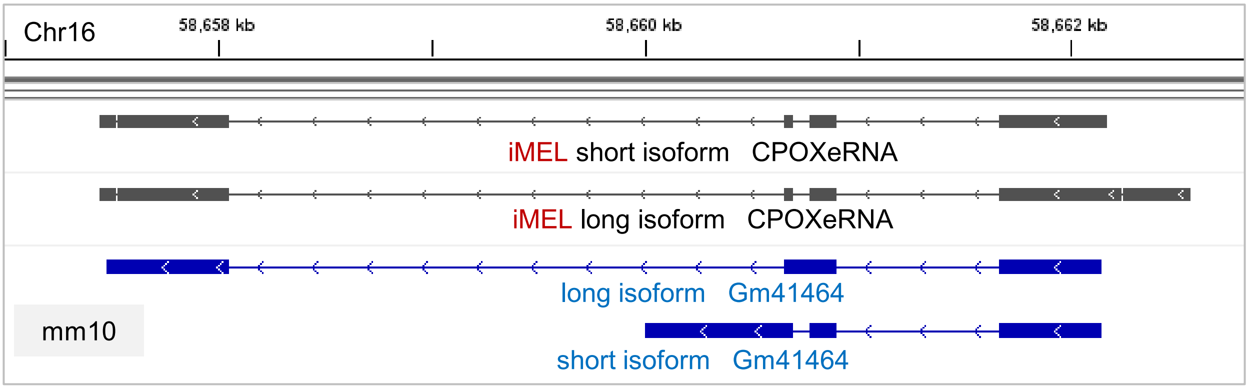

Supplementary Fig.2

A

Isoform	Label	Coding probability	Peptide length(aa)	Fickett score	Isoelectric point
short	noncoding	0.017058	48	0.31855	9.09832763672
long	noncoding	0.0175789	48	0.33006	9.09832763672

B

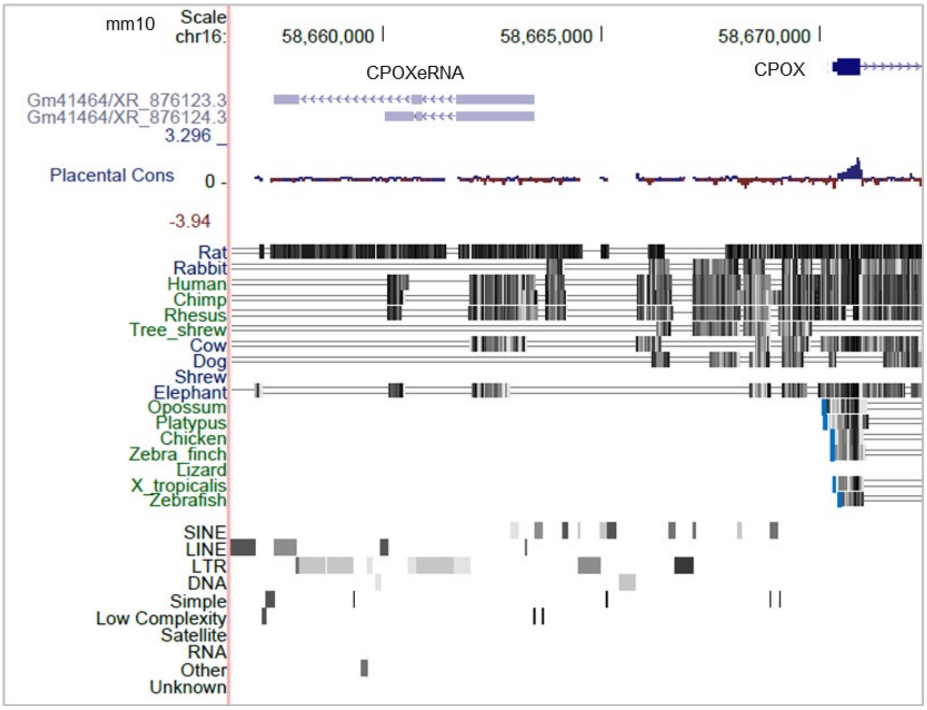

Supplementary Fig.3

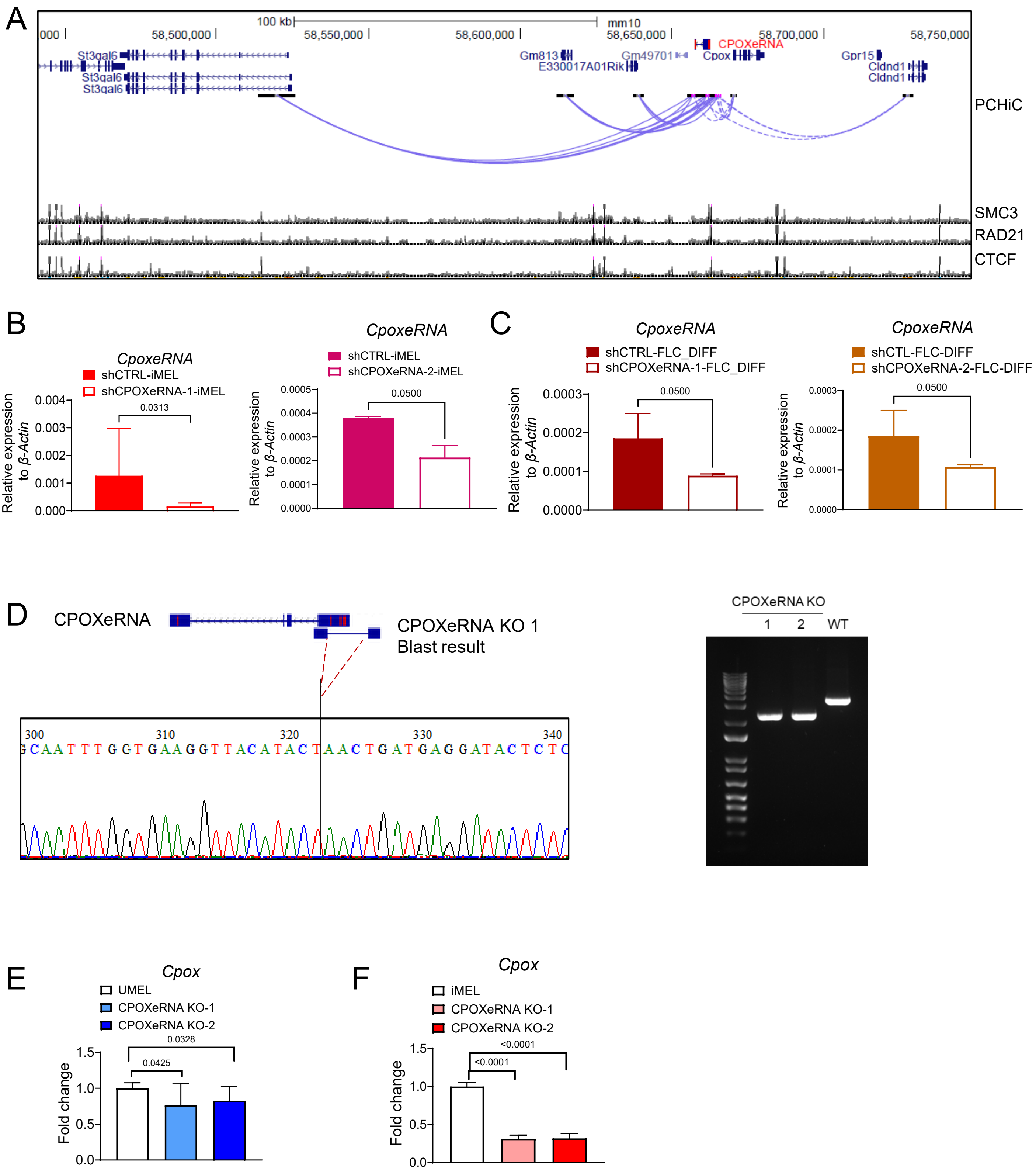

Supplementary Fig.4

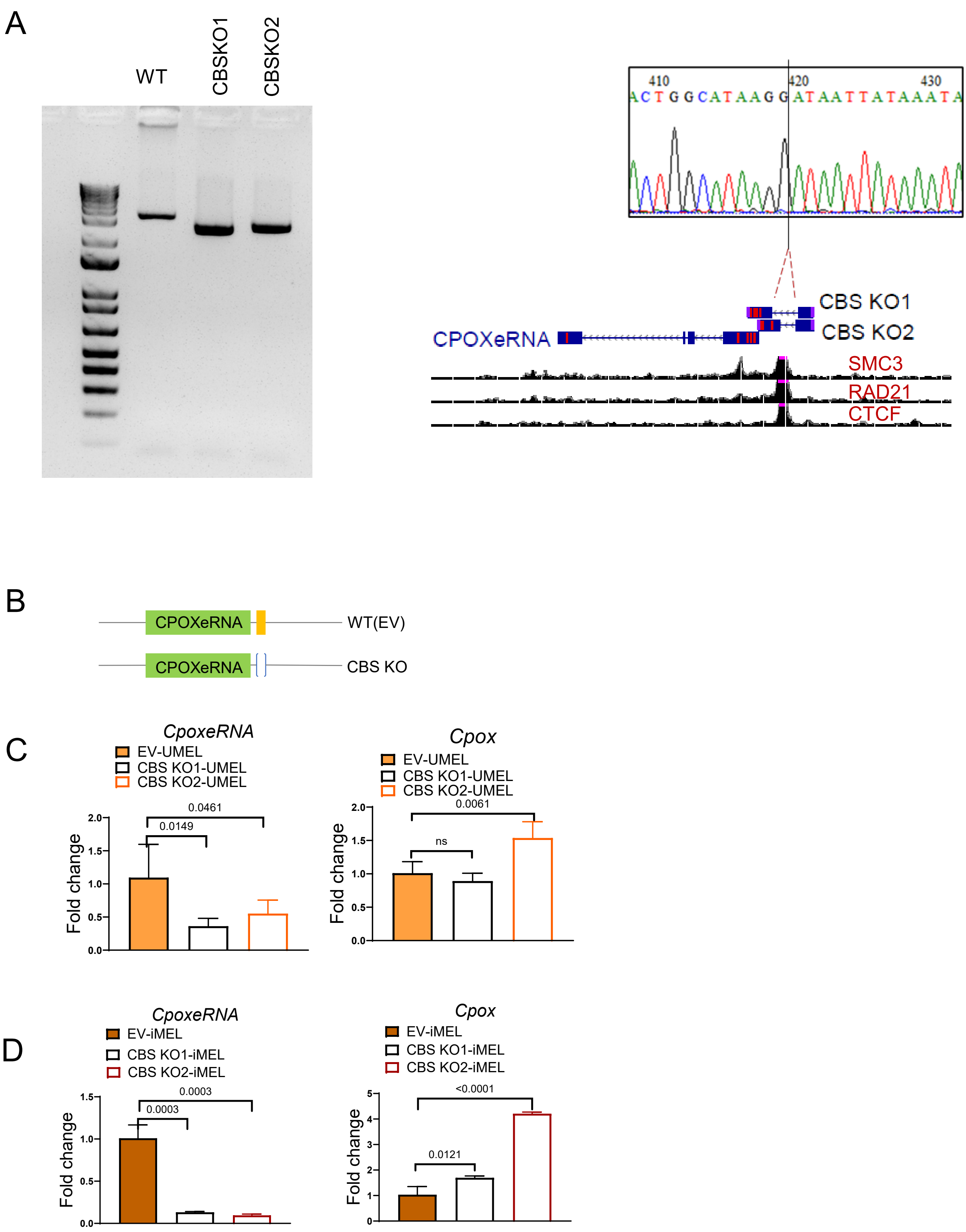

Supplementary Fig.5

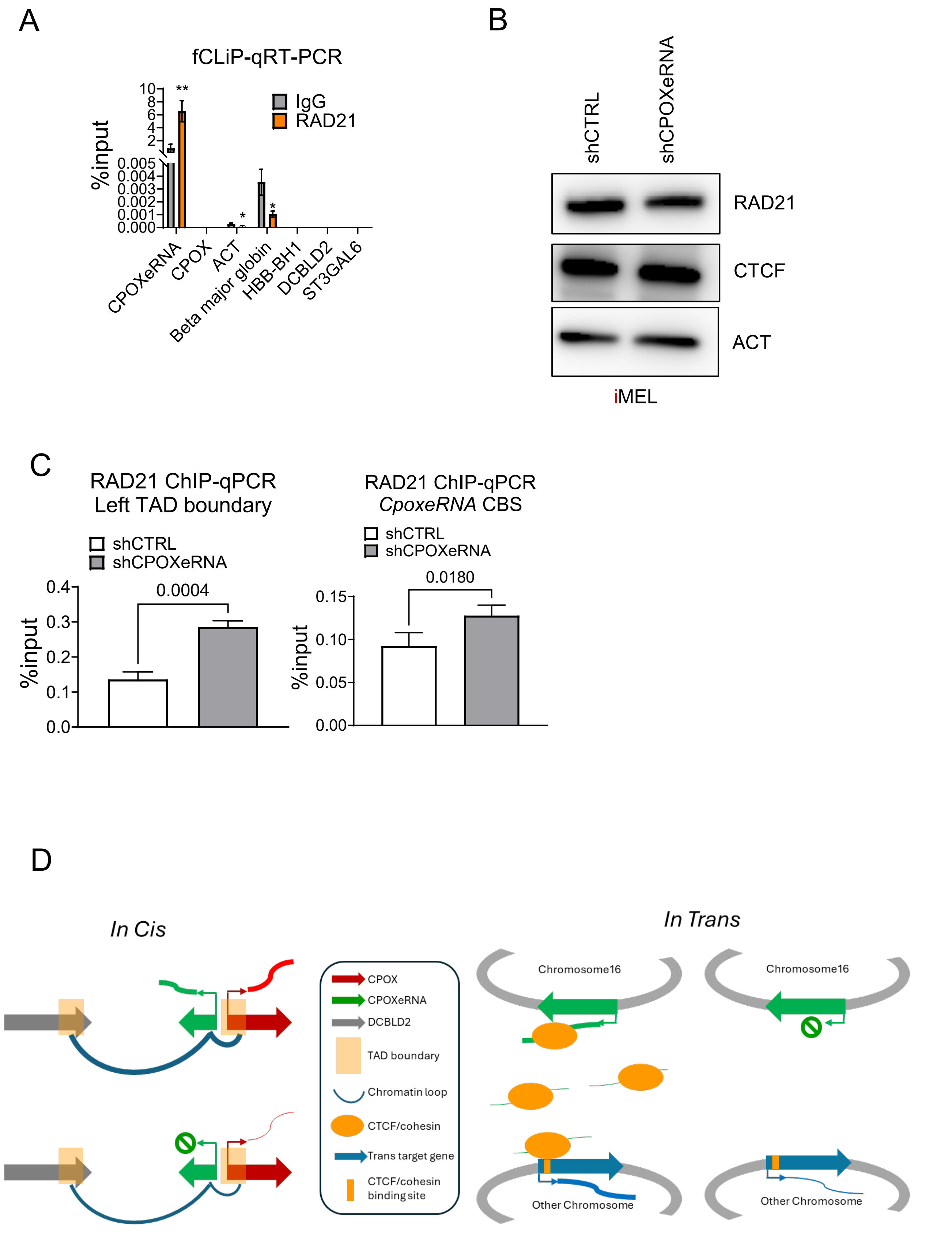

Supplementary Table 1

**shRNA**	
shCPOXeRNA-1	GAATGCTTCCTCCCAACTGAT
shCPOXeRNA-2	GCCAGGCTGAGCTTACCTAAT
**qPCR**	
CPOXeRNA_F	TTCCTCCCAACTGATGAGGATA
CPOXeRNA_R	ATACCTGGCAGCTTCCAGGA
ACT_F	ACCCCATTGAACATGGCATT
ACT_R	TGTAGAAGGTGTGGTGCCAGAT
Gapdh_F	TGCACCACCAACTGCTTAG
Gapdh_R	GGATGCAGGGATGATGTTC
U6 snRNA_F	CCCAGAGATGCAAAGAGGCA
U6 snRNA_R	TTTGCCCTGGTAGGAGCTTG
Cpox_F	CTGAGGAGAGGGCGGTATGT
Cpox_R	TGGCGCAACACTTCCAGAAT
Ercc1-F	CTGGAGACCTACAAGGCGTATG
Ercc1-R	GATTTCACGGTGGTCAGACACTC
Ehmt2-F	GGAGCCAACATCAATGCCGTAG
Ehmt2-R	TAGACAGGTGGAGCCATCCTCT
Hdac7-F	CGCCTCAAACTGGATAACGGGA
Hdac7-R	GCATTGGAGGAATGCAGCTCGT
CPOXeRNA_TCN_F	GCCAGAGATGGGATGAGAGTG
CPOXeRNA_TCN_R	AAGACTCTTCTTTAAGCTGTTTTGT
18S rRNA_F	ATGGTAGTCGCCGTGCCTAC
18S rRNA_R	CCGGAATCGAACCCTGATT
**ChIRP**	
CPOXeRNA_ChIRP_qPCR_F1	CCCCACAAAGAGGTGTCTGA
CPOXeRNA_ChIRP_qPCR_R1	GAGTGCTGGTCCTTCTTGGT
CPOXeRNA_ChIRP_qPCR_F2	GCTTCCCTGACACAGTTCACA
CPOXeRNA_ChIRP_qPCR_R2	ACCTCTTTGTGGGGCTGAAT
mGapdh (promoter)_F(for DNA)	GATTACGGGATGGGTCTGAA
mGapdh (promoter)_R(for DNA)	GCTGCACCTCTGGTAACTCC
mGAPDH F(for RNA)	Tgcccccatgtttgtgatg
mGAPDH R(for RNA)	tgtggtcatgagcccttcc
**ChIRP probes**	
CPOXeRNA_ChIRP_BTEG_1	ctgtttacttgactccagac
CPOXeRNA_ChIRP_BTEG_2	ccaagattttctatcccata
CPOXeRNA_ChIRP_BTEG_3	aaggagcctgcaggattatt
CPOXeRNA_ChIRP_BTEG_4	ggtgtgttctgtgaaaagca
CPOXeRNA_ChIRP_BTEG_5	ctcgggtcagacacttcaaa
CPOXeRNA_ChIRP_BTEG_6	attattctcttcttccctag
**3C**	
DCBLD2 Promoter	GTCCACTACCAAAAGCGCAA
Left TAD boundary	TCACGCTTAGTGGGCAGAGG
ST3GAL6 promoter	CCCAAAAAGCTAACTCTATAACACA
ST3GAL6 gene body	GGCCTTCATTTGAAAATACAGC
CPOXeRNA	TGGAGCTTCCAAGTTCCTCTTG
CPOX promoter	GAATAACCAGTGGCCTCGATTA
CPOX gene body 1	CAGTTTGTTACTGAACACGTCTC
CPOX 3' end	GCAGGGGGCGCAGAATTA
Cldnd1_3C_2_L	AAGGACAGGTACGTCCTTTACG
GM813_3C_1_L	CTGAAGCAATAGAGGAGTAAAAGC
E330017A01Rik_3C_1_L	GCTTTGGAATTCTATGGTGATAGG
**T7 primer**	
CPOX_eRNA_T7_F	gaaatTAATACGACTCACTATAGG AGGATCACATCAATAAGAGGAGGA
CPOX_eRNA_T7_R	TCAGTTCATCATGGCAGGGAA
**sgRNA**	
CPOXeRNA KO-1438	GTGAAGGTTACATACTAGCA
CPOXeRNA KO-1273	GAGTATCCTCATCAGTTGGG
CPOXeRNA_VPR-sgRNA	ATACTGCCCTAGTTCTCGAT
CPOXeRNA_CBS KO-F	GTGAAGGTTACATACTAGCA
CPOXeRNA_CBS KO-R1	TGTATGCATCAGAGCACTTG
CPOXeRNA_CBS KO-R2	ATGCATCAGAGCACTTGTGG
**FISH probes**	
CPOXeRNA_smFISH_msk5_1	ctgtttacttgactccagac
CPOXeRNA_smFISH_msk5_2	ggcaatcttttattgacctg
CPOXeRNA_smFISH_msk5_3	tccaaagattcatccttaga
CPOXeRNA_smFISH_msk5_4	agatctgcataattctagca
CPOXeRNA_smFISH_msk5_5	acatttgtgaggttcaagtt
CPOXeRNA_smFISH_msk5_6	agattttctatcccataaga
CPOXeRNA_smFISH_msk5_7	gactaaacctgttatactcc
CPOXeRNA_smFISH_msk5_8	gggaagctatgtgcaaagtt
CPOXeRNA_smFISH_msk5_9	ttttgcaatgtgaactgtgt
CPOXeRNA_smFISH_msk5_10	taggaaggagcctgcaggat
CPOXeRNA_smFISH_msk5_11	gtggggctgaatgctcaaaa
CPOXeRNA_smFISH_msk5_12	gtggttgcaaagttcagaca
CPOXeRNA_smFISH_msk5_13	ggtgtgttctgtgaaaagca
CPOXeRNA_smFISH_msk5_14	tattaggtaagctcagcctg
CPOXeRNA_smFISH_msk5_15	ctcgggtcagacacttcaaa
CPOXeRNA_smFISH_msk5_16	attattctcttcttccctag
CPOXeRNA_smFISH_msk5_17	gccagaattcatagaagttt
**RACE**	
CPOX_GSPRT_1	GGAAAAGCTGGGCTGCTTTT
CPOX_eRNA_outer_5RACE_2	AAGAAGGACCAGCACTCCTCA
CPOX_eRNA_inner_5RACE_3	CGCCATATGGTGGTTGGCTTTAACAGTGTCC
CPOX_eRNA_outer_3RACE	TGGGGCAGCTCAGTTCATGGAG
CPOX_eRNA_outer_3RACE_3	CACAGCACAATGGGTCAGGAAAC
CPOX_eRNA_outer_3RACE_2	GATGGCCATACGTTTCTTCCAGCT
CPOX_eRNA_3RACE_as3	GAATGCTTCCTCCCAACTGATGAGGAT
CPOX_eRNA_3RACE_as4	TTGTCTGTTTCCTGACCCATTGTGCTGTGAAC
Q_T	ccagtgagcagagtgacgaggactcgagctcaagcttttttttttttttttt
Q_O	ccagtgagcagagtgacg
Q_I	gaggactcgagctcaagc
**ChIP**	
CTCF-CPOXeRNA-F1	CAAAGCCTGCCATTTAGACTCC
CTCF-CPOXeRNA-R1	GTTGCCAATGGGCCTGTGAAT
DCLD2-CTCF-ChIP-Mid-F3	GCTGAGGCCTTTGTGTGATG
DCLD2-CTCF-ChIP-Mid-R3	AGCTAACCTGGCAATACGGG

Supplementary Table 2

Total RNA-seq		
samples	ENCODE ID	ENCODE ID
#SE		
CD8-positivenaiverestingalpha-betaTcellmaleadult	ENCFF017LOD	
T-cell_combined_quant	ENCFF014SLC	
CD4-positive-naive-resting-alpha-beta-T-cell_combined_quant	ENCFF933KQV	
monocyte_combined_quant	ENCFF477YHR	
Bcell_combined_quant	ENCFF248ELC	
neutrophil_combined_quant	ENCFF234LUQ	
layer-of-hippocampus-tissue_combined_quant	ENCFF233IFH	
left-cerebral-cortex-tissue_combined_quant	ENCFF822PGJ	
gastrocnemius-tissue_combined_quant	ENCFF642YTY	
adrenal-gland_combined_quant	ENCFF101BDM	
heart_combined_quant	ENCFF657UWH	
#PE		
G1E	ENCFF302KQL	ENCFF889JPV
CH12.LX	ENCFF001NCT	ENCFF001NCP
megakaryocyte-erythroid-progenitor-cell-male-adult	ENCFF888LGA	ENCFF597XAQ
megakaryocyte-progenitor-cell-male-adult	ENCFF689ZLL	ENCFF168LJP
megakaryocyte	ENCFF894SHM	ENCFF202CQJ
hematopoietic-stem-cell	ENCFF885AJK	ENCFF269OMM
granulocyte-monocyte-progenitor-cell	ENCFF836JVX	ENCFF845MSX
erythroid-progenitor-cell	ENCFF084DCW	ENCFF760PLT
erythroblast	ENCFF389ZWR	ENCFF195KMR
common-myeloid-progenitor	ENCFF048FGD	ENCFF898RXC
midbrain-tissue	ENCFF002FAI	ENCFF002FAJ
liver	ENCFF001RTN	ENCFF001RTM
hindbrain	ENCFF002EZY	ENCFF002EZZ
forebrain	ENCFF002EZU	ENCFF002EZV
MEL	ENCFF001NDZ	ENCFF001NEE
F121-9	ENCFF521IDK	ENCFF006WNS
ES-E14	ENCFF001NDA	ENCFF001NDF
PolyA RNA-seq		
samples	ENCODE ID	ENCODE ID
# SE		
embryo14.5days-liver-tissue	ENCFF635YMK	
embryo14.5days-heart-tissue	ENCFF478ZKL	
embryo14.5days-forebrain-tissue	ENCFF460TCF	
embryo14.5days-hindbrain-tissue	ENCFF275SGM	
embryo14.5days-kidney-tissue	ENCFF021BPG	
embryo14.5days-limb-tissue	ENCFF682WAX	
embryo14.5days-lung-tissue	ENCFF800SJE	
embryo14.5days-midbrain-tissue	ENCFF499UQZ	
embryo14.5days-FacialProminence-tissue	ENCFF316SZZ	
embryo14.5days-NeuralTube-tissue	ENCFF739BEA	
embryo14.5days-brain-tissue	ENCFF001LFW	
spleen	ENCFF001LEU	
embryo14.5days-stomach-tissue	ENCFF775CBB	
embryo14.5days-intestine-tissue	ENCFF904JAW	
thymus	ENCFF001LFN	
adrenal-gland-tissue	ENCFF867HND	
cerebellum-tissue	ENCFF001LBM	
small-intestine-tissue	ENCFF001LFE	
testis-tissue	ENCFF001LFB	
cortical-plate-tissue	ENCFF001LBT	
gonadal-fat-pad-tissue	ENCFF001QMU	
placenta	ENCFF001LEQ	
skeletal-muscle-tissue	ENCFF642EVR	
urinary-bladder-tissue	ENCFF603WRT	
Bcell	ENCFF001QJS	
embryonic-fibroblast-primary-cell	ENCFF001LDS	
naive-thymus-derived-CD4-positive-alpha-beta-T-primary-cell	ENCFF001QVU	
NIH3T3	ENCFF001QSM	
CH12.LX	ENCFF001MFQ	
A20	ENCFF001QKU	
416B	ENCFF001QIU	
Patski	ENCFF024WIO	
G1E	ENCFF001MGT	
MEL	ENCFF001QRQ	
ES-Bruce4	ENCFF001LCA	
telencephalon	ENCFF001QLZ	
embryo	ENCFF001QPO	
olfactory-bulb	ENCFF001LED	
#PE		
embryo-14days-central-nervous-system-tissue	ENCFF001IOG	ENCFF001IOH
ovary	ENCFF463WEH	ENCFF312OKA
embryo-14.5days-megakaryocyte-primary-cell	ENCFF001MKH	ENCFF001MKP
macrophage	ENCFF198GMP	ENCFF075DXA
leukemia-stem-primary-cell	ENCFF001MGJ	ENCFF001MGM
erythroblast-primary-cell	ENCFF001MGD	ENCFF001MGA
G1E-ER4	ENCFF001MJW	ENCFF001MKB
C2C12	ENCFF001IDZ	ENCFF001IDY
C3H10T12	ENCFF001ICI	ENCFF001ICJ
adipose-tissue	ENCFF128KGA	ENCFF510DLJ
colon	ENCFF001IPQ	ENCFF001IPW
duodenum	ENCFF001IRZ	ENCFF001ISG
frontal-cortex-tissue	ENCFF001IUD	ENCFF001IUF
layer-of-hippocampus-tissue	ENCFF083WGC	ENCFF784VYU
left-cerebral-cortex-tissue	ENCFF814YLU	ENCFF858ZSD
mammary-gland-tissue	ENCFF001JGE	ENCFF001JGK
pancreas	ENCFF849WWH	ENCFF224IMT
subcutaneous-adipose-tissue	ENCFF001JKF	ENCFF001JKJ
